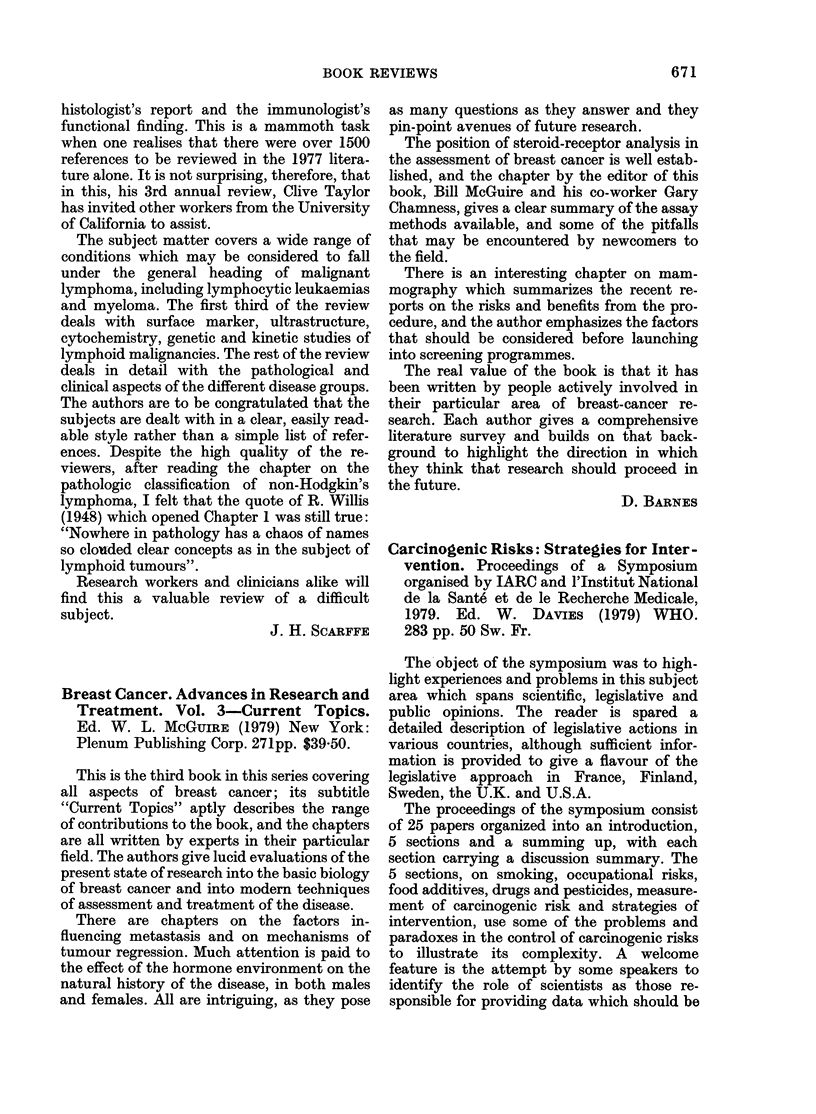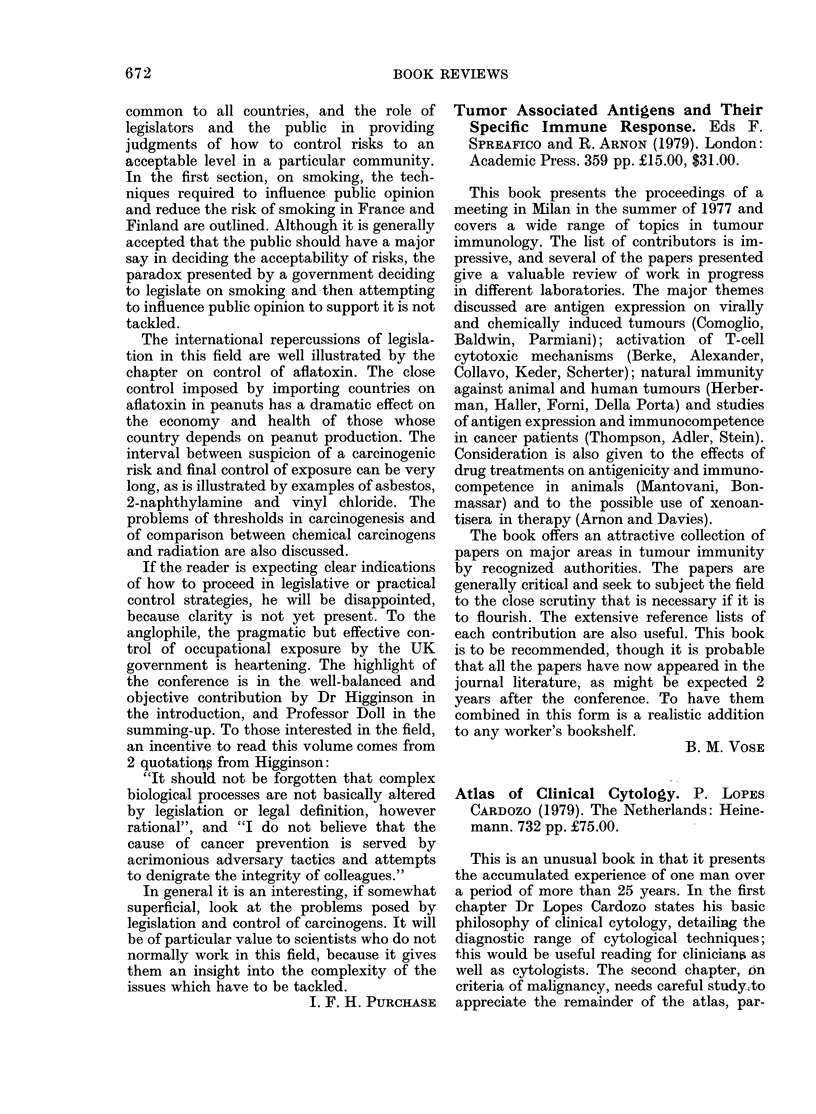# Carcinogenic Risks: Strategies for Intervention

**Published:** 1980-04

**Authors:** I. F. H. Purchase


**Carcinogenic Risks: Strategies for Intervention.** Proceedings of a Symposium organised by IARC and l'Institut National de la Santé et de le Recherche Medicale, 1979. Ed. W. Davies (1979) WHO. 283 pp. 50 Sw. Fr.

The object of the symposium was to highlight experiences and problems in this subject area which spans scientific, legislative and public opinions. The reader is spared a detailed description of legislative actions in various countries, although sufficient information is provided to give a flavour of the legislative approach in France, Finland, Sweden, the U.K. and U.S.A.

The proceedings of the symposium consist of 25 papers organized into an introduction, 5 sections and a summing up, with each section carrying a discussion summary. The 5 sections, on smoking, occupational risks, food additives, drugs and pesticides, measurement of carcinogenic risk and strategies of intervention, use some of the problems and paradoxes in the control of carcinogenic risks to illustrate its complexity. A welcome feature is the attempt by some speakers to identify the role of scientists as those responsible for providing data which should be common to all countries, and the role of legislators and the public in providing judgments of how to control risks to an acceptable level in a particular community. In the first section, on smoking, the techniques required to influence public opinion and reduce the risk of smoking in France and Finland are outlined. Although it is generally accepted that the public should have a major say in deciding the acceptability of risks, the paradox presented by a government deciding to legislate on smoking and then attempting to influence public opinion to support it is not tackled.

The international repercussions of legislation in this field are well illustrated by the chapter on control of aflatoxin. The close control imposed by importing countries on aflatoxin in peanuts has a dramatic effect on the economy and health of those whose country depends on peanut production. The interval between suspicion of a carcinogenic risk and final control of exposure can be very long, as is illustrated by examples of asbestos, 2-naphthylamine and vinyl chloride. The problems of thresholds in carcinogenesis and of comparison between chemical carcinogens and radiation are also discussed.

If the reader is expecting clear indications of how to proceed in legislative or practical control strategies, he will be disappointed, because clarity is not yet present. To the anglophile, the pragmatic but effective control of occupational exposure by the UK government is heartening. The highlight of the conference is in the well-balanced and objective contribution by Dr Higginson in the introduction, and Professor Doll in the summing-up. To those interested in the field, an incentive to read this volume comes from 2 quotations from Higginson:

"It should not be forgotten that complex biological processes are not basically altered by legislation or legal definition, however rational", and "I do not believe that the cause of cancer prevention is served by acrimonious adversary tactics and attempts to denigrate the integrity of colleagues."

In general it is an interesting, if somewhat superficial, look at the problems posed by legislation and control of carcinogens. It will be of particular value to scientists who do not normally work in this field, because it gives them an insight into the complexity of the issues which have to be tackled.

I. F. H. Purchase